# COVID-19 Vaccine Hesitancy in Trinidad and Tobago: A Qualitative Study

**DOI:** 10.7759/cureus.43171

**Published:** 2023-08-08

**Authors:** Shastri Motilal, Daina Ward, Kymera Mahabir, Thea Lopez, Raesha Logan, Shastri Maharaj, Jenair Maloney, Monique Marson, Chadé Marcelle

**Affiliations:** 1 Paraclinical Sciences, The University of the West Indies at St. Augustine, St. Augustine, TTO

**Keywords:** trinidad and tobago, motivation factors, fear of covid19, trust in government, qualitative research

## Abstract

Background

After three years of COVID-19, the WHO declared that the pandemic was no longer a global health emergency. Vaccination remains part of the management strategy, given the current phase of the pandemic. This study explored the reasons for COVID-19 vaccine hesitancy in Trinidad and Tobago (TT).

Methodology

A qualitative study of persons 18 years and over from the eastern, northwest, northcentral, and southwestern geographical areas of TT, who are unvaccinated and hesitant, was done by convenience sampling. Formal in-depth virtual interviews were done on a one-to-one basis using a semi-structured questionnaire. The interviews were recorded and transcribed using the principles of reflexive thematic analysis of participants' responses.

Results

From 25 participants' responses, the main themes for being vaccine-hesitant were fear, inefficacy, information inadequacy, perceived susceptibility, mistrust, herbal alternatives, and religious hesitations. Additionally, their motivations for receiving the vaccine in the future were surrounded by themes of necessity, perceived susceptibility, health benchmark, and assurance.

Conclusion and recommendations

This qualitative investigation identified traditional factors contributing to COVID-19 vaccine hesitancy and unique determinants such as herbal use and religious beliefs within the TT context. These insights could inform future research and facilitate the development of tailored strategies to address persistent vaccine hesitancy for COVID-19.

## Introduction

As of July 2023, there have been over 700 million confirmed cases of COVID-19 globally, with almost 7 million deaths [1]. Trinidad and Tobago (TT) has not been spared, with over 191,000 confirmed cases and more than 4,300 deaths in July 2023 [2]. Geographically, TT is in the southern Caribbean and is a twin island state with a population of approximately 1.5 million [3]. Despite the availability of COVID-19 vaccines in TT since April 2021, the percentage of persons in May 2023 who completed a vaccination regimen stood at approximately 50% [4,5]. This was below the anticipated 63% of those who expressed willingness to take the shot before COVID-19 vaccines became available in TT [6]. Two studies have been done for healthcare professionals in TT documenting good COVID-19 knowledge, positive attitudes, and perceptions [7,8]. Reasons for vaccine hesitancy among TT primary care workers were fear of adverse effects, lack of information, and inadequate duration of trials [7]. In the study of TT dentists, about one-tenth of respondents expressed safety concern, and two-fifths were worried about side effects. A TT study in pregnant women revealed a COVID-19 vaccine uptake of 24%, with most women expressing fear that the vaccine would harm their babies or that insufficient data was available [9]. In May 2023, the WHO determined that COVID-19 was no longer a public health emergency of international concern, and the integration of COVID-19 vaccines as part of life course programs was amongst its recommendations [10]. This underscores the importance of COVID-19 vaccination even in the devolving stage of the pandemic, particularly for high-risk groups. The low COVID-19 vaccination coverage rates seen in TT can be due to various factors unique to this setting contributing to vaccine hesitancy. The local studies highlighted above were cross-sectional by design. This research aimed to explore the reasons for vaccine hesitancy in the TT population.

## Materials and methods

Study design, setting, and sampling

This qualitative study was conducted in TT from March to May 2022, a transition time when governmental restrictions on social gatherings, mask-wearing, and travel requirements were relaxed [11]. With fewer than 50% of the population vaccinated during this time, the unvaccinated population served as the main group from which participants were drawn. Contacts of the authors, residing in the four main regions of TT, were recruited through convenience sampling using personal correspondences and social media. Included in the study were adults who had not received the COVID-19 vaccine and were hesitant to do so, along with their informants. Fully vaccinated individuals, those willing to obtain the vaccine soon, and vulnerable populations, including children, prisoners, and mentally disabled persons, were excluded from participation.

Data collection, analysis, and validation

Given the restrictions present at the time and the hesitancy of respondents to meet face-to-face, individual virtual interviews were conducted to explore vaccine hesitancy in T&T. The semi-structured questions asked were: 1) 'Tell me about your reasons for being unwilling to receive the COVID-19 vaccine.' 2) 'Would you ever consider becoming vaccinated in the future? (If yes) What would make you more open to receiving the COVID-19 vaccine in the future?' These questions were developed by the researchers after reaching a consensus based on their informal discussions with personal vaccine-hesitant contacts. Audio recordings of the interview sessions were made by each interviewer using the Blackboard Collaborate Ultra web conferencing system, available to the researchers through their institution. The interview was recorded automatically online from the start to the end of the conferencing call for the duration of the interview. All audio recordings were later transcribed verbatim using Otter.ai software [12].

The analysis of data was done using the principles of reflexive thematic analysis using an inductive approach. For initially apparent omissions or errors, transcripts were reviewed together with the matching audio recording. The data was anonymized and recorded using participant numbers in Word (MS Office). The research team collectively read the transcripts to gain familiarity with the data and a broad understanding of the content. Independent researchers organized each transcript into codes in Excel (MS Office), with the senior researcher reviewing them. Preliminary themes were derived by individual researchers, and consensus meetings were held to review and refine the themes. Participant validation was used, wherein respondents were asked to review the transcripts and themes for agreement and refinement. Recruitment was halted when saturation occurred from the ongoing analysis of the data. Themes were tabulated with supporting quotations.

Ethical considerations

Participant consent was obtained prior to the interviews via an online form that guaranteed the collection and storage of anonymized data. Ethics approval was obtained from The University of the West Indies Ethics Committee (Ref: CREC-SA.1389/02/2022).

## Results

The characteristics of the participants are shown in Table 1. There were 13 females and 12 males, ages 20-83, from both rural and urban regions on both islands.

**Table 1:** Demographics of participants and identifiers

Participant No.	Age (years)	Gender	Geographic area
1	39	F	St. Helena
2	35	M	Las lomas
3	58	M	Charlieville
4	77	F	Caanan
5	83	F	Port of Spain
6	71	M	Mt. Pleasant
7	57	F	Laventille
8	66	F	Maloney
9	66	M	St. James
10	20	F	San Juan
11	52	M	Union Hall
12	37	F	Gasparillo
13	36	F	San Fernando
14	52	F	Gasparillo
15	22	M	Marabella
16	27	F	Point Fortin
17	21	F	Gasparillo
18	20	M	Gasparillo
19	26	F	San Juan
20	31	M	Arima
21	78	M	Mausica
22	29	M	Couva
23	24	M	Port of Spain
24	23	M	Port of Spain
25	23	F	St. Augustine

Reasons for not getting COVID-19 vaccine

In this study, several themes emerged as to why participants were hesitant to be vaccinated against COVID-19. These themes were: fear, inefficacy, information inadequacy, perceived susceptibility, mistrust, better alternatives, and religious hesitations (Table 2).

**Table 2:** Themes of reasons for COVID-19 vaccine hesitancy with sub-themes and quotations.

Theme	Sub-theme	Supporting quotation
Fear	Safety in Pregnancy	“And next thing that is a next concern is pregnant women taking COVID-19 vaccines because to me like when you're pregnant, you hardly can take anything, can’t take a tablet, you can’t take certain things...” [Participant no. 19]
	Fear of Death from the Vaccine	“From personal experience, a relative of mine took the vaccine. God help his family. He dropped down dead right after the shots.” [Participant no. 21]
	Medical Concerns	“I have 14 Medical challenges. And I don't think I'm a candidate for that vaccine yet until it is very, very safe. Perhaps not 100% but very very very safe. Only then and then I would take a vaccine because I had six heart attacks, I'm a diabetic. Hypertension, prostate, osteoporosis, arthritis, and other lots of other medical challenges.” [Participant no. 21]
	Fear of Needles	“I have a phobia for needles.” [Participant no.16]
	Adverse Effects	“I think the risk, uh…firstly, in women because I’ve heard from people, close family members that they have a change in their cycle, they bleed more, it lasts longer, it is…it comes twice a month. So, it’s messed up their entire menstrual cycle and I don’t like that…” [Participant no.25]
Inefficacy		“I really don't know if you take or you don't take it or not if it will make a difference. I know somebody who was fully vaccinated and the person died…... whether you take it or you don’t take it, it’s not saving you” [Participant no. 25]
Information inadequacy	Too much information	“an excess overload of information…From some people, they saying that you should take the vaccine...good for you, and then on a next side...it have people who bring up the fact that the vaccine doesn't work for everybody” [Participant no. 18]
	More research needed	“It is not safe 100%...like the tetanus, polio, the bacteria, those vaccination those definitely safe…because years and years of research went into those vaccines. They use animals for many many years. And then they started using humans. With this COVID-19 vaccine…Instead of using animals they started using humans as guinea pigs to do the experiment.” [Participant no. 21]
	Lack of information	“I feel like information concerning side effects is not so public… like they don't make it as available because they keep it on a hush hush…You will more hear about taking the vaccine, but you don't hear the side effect that is happening in Trinidad…you will hear okay nobody really has side effects.” [Participant no. 19]
Perceived susceptibility	Natural Immunity	“We also know that we could fight it naturally, because that is what our bodies are built for. I think this thing with children and vaccinating children under five and I, I can't even wrap my head around that I wouldn’t even go there.” [Participant no.1]
	Unnecessary	“Well, my current reason is that I'm home most of the times and yeah, so I didn't really want to get it because I don't go out that much.” [Participant no. 17]
	COVID Infection is Mild	“I know children have got it in the family as well for children a baby two and a half years old. He got it and he was fine. He just had a little fever and he fight through that like nothing and he was good.” [Participant no.1]
	Pandemic is Temporary	“Pandemics in the past come and go… So, I know it’s only a matter of time.” [Participant no. 10]
Mistrust	Vaccine's Contents	“And two, the ingredients within the uh… the vaccination is not being displayed, it's being hidden or is not being answered by any doctors and three side effects from the World Health.” [Participant no. 22]
	Vaccine's Manufacturers’	“... I have seen for myself how, you know, like just taking simple things as tablets and stuff does make you get dependent on pharmaceuticals and the pharmaceutical industry is a multi-billion-dollar business. And that’s hence the reason why things like AIDS and ca- well like AIDS, I wouldn't [unintelligible] to cancer, but like AIDS, they wouldn't bring out the cure because they would make more money…" [Participant no. 10]
	Experimental Treatment	“I don't think it's a vaccine. I will call it an experimental trial of a…well you can't say a vaccine but a…jab or something, okay.” [Participant no. 3]
	Fast Development of Vaccine	“One of the concerns as well is the period in which the vaccine was created. I understand that we have technology and technology could make these vaccinations quickly, but what about the trial period? So I am thinking that a vaccine trial period is a couple years so COVID-19 vaccines came out so fast.” [Participant no. 19]
	Unsure of Safety	“There are a lot of controversy worldwide concerning this vaccine. Many people are saying even scientists even doctor, medical expert saying it is definitely not 100% safe and some others saying it is safe.” [Participant no. 21]
Herbal alternatives		“I decided to go with herbs. Herbs like ginger, turmeric, lemongrass and other herbs. That is the reason why I am not taking the vaccine”. [Participant no. 4]
Religious Hesitations		“I feel comfortable in everything. I am just not spiritually moved yet. I'm not saying that I don't want to. It just hasn’t come to me to do it yet.” [Participant no. 5]

Motivations for receiving COVID-19 vaccine in the future

Based on the plethora of information received from participants, there were many reasons why they would be motivated to take the COVID-19 vaccine in the future. The main themes arising from these motivations were: necessity, perceived susceptibility, health benchmark, and assurance. Table 3 summarizes these themes with sub-themes and quotations.

**Table 3:** Themes of motivation for future COVID-19 vaccine uptake.

Theme	Sub-theme	Supporting quotation
Necessity	Only if absolutely necessary	“...because they…well I mean if they remove all the restrictions…. nah! I will wing it till death. But I mean, if they keep it the way it is that we don’t have access to particular things then I'll probably go for long I could go for, but I not… wouldn’t be hesitant to taking it but if I really need it for something.” [Participant no. 23]
		“If I had to on my own, I will not take the vaccine. But, due to how the world is operating right now, I think that I would have to take the vaccine. Why? Everything is, safe zone, if you’re not vaccinated you can’t do this, you can’t do that. So, it kind of…it appears like you’re being forced to take this vaccine and I don’t really like it. But I think because of how everything is now, I may have to do it….” [Participant no. 25]
	Requirement	“Yeah, if I need it to do something I'll take it. Honestly, nothing really. I just never was put in a situation where I needed to take it so I didn't take it.” [Participant no. 18]
Perceived susceptibility	Fear of contracting virus	“... the job I do at public transport interacting within the buses making it clear right so, that is one of the reasons why I knew I will have to take it…” [Participant no. 8]
	Fear of death without having the vaccine	“my brother died from COVID and he wasn’t vaccinated” [Participant no. 7]
Health Benchmark		“I think I’ll need to upkeep a very healthy lifestyle. Where I exercise often, I have a whole diet change, I keep active… to keep my body, you know, where it should be... I need to change my whole lifestyle around if I do receive this vaccine.” [Participant 25]
Assurance	Need for surety	“So for me to take the vaccine, I think I have to wait a few years to see the side effects and see what's going on with other people before I decide to put it in my body because I'm not sure how my body will react.” [Participant no. 19]
	No adverse effects	“Yes, I would just prefer that you can get vaccinated with no worries about possibly dying from any kind of side effect or anything like that because the vaccine supposed to prevent you from dying and if not then it not really worth it…” [Participant no. 7]
	More transparency	“unless they can display what's in inside any vaccine” [Participant no. 22]
	Experience of others	“I believe it’s just a mindset thing because my brother you know every time we talk he always mentions it to me try to make me feel you know really comfortable about it because this is not for man you know it’s really for God and he always tries his best to explain that whether I fear it or not there will be a time I have to take it.” [Participant no. 8]
	More research needed	“If a lot more research was done on the vaccine where…it has less side effects and less deaths and…the vaccine was actually tested just like a tetanus vaccine...the tetanus vaccine…does not stop you from getting a nail stick…but it will protect you from tetanus itself. So, unless it reaches to a stage where the vaccine actually proved to be valid and it working, efficiently, then only then I would consider entertaining the thought of taking the covid vaccine.” [Participant no. 2]

## Discussion

In this TT study, participants expressed various reasons for being hesitant about the COVID-19 vaccine. Most of the themes identified in this study were also identified in the systematically reviewed literature [13]. A thematic synthesis on qualitative research surrounding COVID-19 vaccine hesitancy described themes of "institutional mistrust," "lack of confidence in vaccine and vaccine development process," "lack of reliable information or messengers," "complacency/perceived lack of need," and "structural barriers to vaccine access" [14]. These overlap with the themes of inefficacy, information inadequacy, perceived susceptibility, and mistrust, as seen in this study. This study generated sub-themes of concerns surrounding death and adverse effects from the vaccine, safety in pregnancy, and effects on pre-existing medical illnesses. This overarching theme of fear was highlighted in a study that examined the fearful impact of the arrival of the COVID-19 vaccine on a global scale [15]. Vaccine novelty, misinformation, and uncertainty are possible explanations for this effect that were also described in this study. Several interventions have been explored for reducing COVID-19 vaccine hesitancy. Most observational studies have described the benefits of appointment reminders, opt-out scheduling systems, multi-modal interventions, and infographics [16]. The themes identified in this study are amenable to such tactics. While the vaccine hesitancy themes identified are not unique to our setting, interventions for these factors can be contextualized to the local setting. TT is a very multicultural society, with several religions being subscribed to [17]. Religiosity and its positive correlation with life satisfaction have been demonstrated in the Trinidadian context [18]. However, when it comes to COVID-19 vaccination, spirituality has been negatively correlated in various settings globally [19]. A regional Jamaican study, however, found that support from religious leaders reduced COVID-19 vaccine hesitancy [20]. This speaks to the potential influence that spiritual factors can have in promoting or discouraging behavior. Involving TT's multiple religious bodies in future vaccine uptake efforts will be key to reducing vaccine hesitancy. Another theme that arose in this study was that of herbal use. The perception that complementary medicines can be protective against COVID-19 was depicted in this study. While the data on herbal options to prevent COVID-19 infection appear mostly theoretical, others have called for research into locally available natural compounds [21,22]. TT has a history of many well-documented naturally occurring remedies, some of which are more popularly used than others [23,24]. Anticipatory guidance, given our familiarity with the natural remedies being used in TT, may help reduce misinformation where mindsets exist about herbs as an alternative to vaccine-induced immunity [25]. Though participants of this study were vaccine-hesitant, they were not opposed to the idea of getting vaccinated in the future. Motivating factors were necessity, perceived susceptibility, need for assurance, and health benchmark. The notion of having to attain ideal health before receiving the COVID-19 vaccine was seen in our study. Fear of receiving the vaccine due to ill health was also voiced. It was interesting that the susceptible persons felt medical illness disqualified them from vaccination when, in fact, they are the high-risk group who may stand to benefit the most. Dissonance-based interventions have been suggested by some as a means to counteract such misperceptions [26]. Vaccine education that is personalized to the local context, based on qualitative work, does have a role in producing tailored interventions [27]. A strength of this work was the diverse demographics of the participants spanning a wide age group and diverse geographic locations. Member checking also added to the credibility of the findings. This study uncovered nuanced findings surrounding local herbal and religiosity factors as they relate to COVID-19 vaccine hesitancy. There are some limitations to this work. Focus group interviews were initially intended for gathering data in this qualitative study. However, during the recruitment process, we found that several participants expressed discomfort in sharing their views in the presence of others, even if their identities were kept anonymous. As a result, it was necessary to adjust our approach and utilize online one-to-one interviews. This approach still allowed us to explore the complex perceptions, attitudes, and beliefs surrounding the topic. While we also conducted online interviews, face-to-face interaction might have enhanced communication and the quality of information shared; however, this was not possible given the state of restrictions at the time of the study. Lastly, it is possible that individuals or groups with unique vaccine-hesitant factors remain unexplored by this study. We did not use stratified sampling, which could have captured a greater diversity of subgroups for exploration, such as pregnant women and health care providers.

## Conclusions

This qualitative study delved into the reason for COVID-19 vaccine hesitancy. While it reaffirmed common global factors like concerns about vaccine safety and mistrust in healthcare institutions, it also unveiled unique elements contributing to vaccine hesitancy in TT. Using herbal remedies and strong religious beliefs emerged as factors with local underpinnings. The herbal culture and faith-based attitudes present unique challenges to vaccine uptake within this context. These findings not only contribute to a more profound understanding of vaccine hesitancy but also pave the way for further research. By recognizing these factors, tailored vaccine uptake campaigns can be developed, catering to the specific needs and beliefs of the TT population. As the world continues to grapple with COVID-19, alongside other infectious diseases, understanding and addressing the roots of vaccine hesitancy, remain crucial.
